# The Role of Greek Olive Leaf Extract in Patients with Mild Alzheimer’s Disease (the GOLDEN Study): A Randomized Controlled Clinical Trial

**DOI:** 10.3390/neurolint16060095

**Published:** 2024-10-29

**Authors:** Sofia Loukou, Georgia Papantoniou, Anastasia Pantazaki, Magdalini Tsolaki

**Affiliations:** 1 1st Department of Neurology, Medical School, “AHEPA” General Hospital Medical School, Faculty of Health Sciences, Aristotle University of Thessaloniki, Makedonia, 54124 Thessaloniki, Greece; tsolakim@auth.gr; 2 Greek Association of Alzheimer’s Disease and Related Disorders—GAADRD, 54124 Thessaloniki, Greece; 3 Laboratory of Neurodegenerative Diseases, Center for Interdisciplinary Research and Innovation (CIRI-AUTH), Balkan Center, Aristotle University of Thessaloniki, 54124 Thessaloniki, Greece; gpapanto@uoi.gr (G.P.); natasa@chem.auth.gr (A.P.); 4 Laboratory of Psychology, Department of Early Childhood Education, School of Education, University of Ioannina, 45110 Ioannina, Greece; 5 Laboratory of Biochemistry, Department of Chemistry, Aristotle University of Thessaloniki, 54124 Thessaloniki, Greece

**Keywords:** Alzheimer’s disease, olive leaf extract, Mediterranean diet, mild dementia, natural compounds

## Abstract

**Background**: Olive leaves are a significant source of biophenols, which have a beneficial impact on cognitive performance. **Objective**: To examine, for the first time, in humans the effect of the daily consumption of a beverage containing olive leaf extract (OLE) versus a Mediterranean diet (MeDi) on patients diagnosed with mild Alzheimer’s Disease (AD), in addition to their regular treatment. **Methods**: A randomized clinical trial compared OLE’s effects on cognitive and functional performance in 55 mild AD patients. Each participant was randomly assigned to two groups: (1) Group 1 was given olive leaves for making a daily beverage and MeDi instructions through monthly diet programs; (2) Group 2 received only the MeDi instructions. After six months, all participants underwent a second neuropsychological evaluation. **Results**: Group 1 participants had statistically significantly higher MMSE scores compared to Group 2 with a *p*-value of 0.0135. Specifically, the mean MMSE difference in patients receiving OLE was close to 0, indicating no memory deterioration, whereas in controls it was −4.1, indicative of cognitive decline. The remaining neuropsychological assessments (FRSSD, FUCAS, ADAS-Cog, CDR, GDS, and NPI) revealed better results in the OLE group, except for GDS, which showed no change, but without statistically significant differences between the two groups.

## 1. Introduction

Dementia is a progressive neurological disorder that deteriorates cognitive abilities, resulting in challenges with daily activities, including financial management, transportation, and personal care. Common symptoms encompass memory impairments, disorientation, and difficulties with concentration and attention [1]. Alzheimer’s Disease (AD) is the predominant type of dementia, initially recognized by Alois Alzheimer in 1906. Despite more than a century of investigation, the exact causes of AD remain contentious. The predominant hypothesis focuses on the aggregation of amyloid beta (Aβ) plaques, believed to propel disease advancement. Nonetheless, alternative mechanisms, such as tau protein hyperphosphorylation, oxidative stress, and neuroinflammation, are also thought to play a role in the pathogenesis of AD [2]. Genetic factors significantly influence the development of dementia, especially mutations in the amyloid precursor protein (APP) and presenilin 1 and 2 genes. Additional risk factors encompass an increasing age, the female gender, low levels of education, the presence of ApoE ε4 alleles, and lifestyle-related elements, including physical inactivity, diabetes, cardiovascular disease, smoking, and hypertension [3,4].

Current pharmacological treatments for AD primarily target symptoms rather than the underlying disease process. Cholinesterase inhibitors, such as donepezil, rivastigmine, and galantamine, are commonly prescribed for patients in the early-to-moderate stages of the disease. These agents increase acetylcholine levels in the brain, temporarily improving cognitive function and reducing behavioral symptoms. Memantine, an NMDA receptor antagonist, is used in moderate-to-severe cases, either alone or in combination with cholinesterase inhibitors. However, these treatments offer only modest benefits and do not halt disease progression [5]. Furthermore, therapies targeting the amyloid hypothesis have been largely unsuccessful. Strategies aimed at reducing Aβ production through the inhibition of β or γ secretase or promoting Aβ clearance via immunotherapy (active vaccines or passive administration of anti-amyloid antibodies) have shown limited efficacy in clinical trials. Recently, anti-amyloid antibodies, such as lecanemab, have shown some ability to slow disease progression, though their clinical benefits remain modest, and they carry risks of adverse effects [6,7].

Given the limitations of conventional treatments, there is growing interest in natural products as alternative therapies for AD and other chronic diseases. Olive leaves, in particular, have been studied for their bioactive compounds, which include polyphenols, like oleuropein and hydroxytyrosol. These compounds exhibit antioxidant, anti-inflammatory, and neuroprotective properties, making them promising candidates for treating age-related diseases [8,9].

In animal studies, olive leaf extract (OLE) has been associated with weight loss, a decreased fat cell size, reduced hyperglycemia and dyslipidemia, and a lower risk of metabolic syndrome [10,11,12]. Human studies indicate that oleuropein improves glucose metabolism, lowers HbA1c, and reduces fasting plasma insulin levels [13,14]. Moreover, clinical studies indicate that OLE can significantly lower blood pressure, yielding results akin to antihypertensive drugs, while also favorably affecting arterial stiffness and decreasing total cholesterol, LDL, and triglyceride levels [15,16,17,18,19]. Olive polyphenols also exhibit anti-cancer properties. In vitro and in vivo studies have demonstrated that olive polyphenols inhibit cancer progression by reducing inflammation and modulating molecular pathways [20]. A review of 25 studies found that high olive oil intake was associated with a 38% reduction in breast cancer risk and a decreased incidence of cancers in the upper aerodigestive tract [21]. Additionally, olive polyphenols have a structure similar to estrogens, meaning they can interact with estrogen receptors and potentially decrease the prevalence and progression of hormone-related cancers, such as breast and prostate cancer [22]. The antioxidant and anti-inflammatory properties of biophenols make them promising candidates for autoimmune disease treatments as well. Studies show that oleuropein and hydroxytyrosol reduce neutrophil infiltration, downregulate pro-inflammatory cytokines like TNF-a and reactive oxygen species (ROS), and promote anti-inflammatory pathways [11,23,24,25,26]. These effects have been observed in models of rheumatoid arthritis and ulcerative colitis [27,28,29]. Olive leaf extract also exhibits antimicrobial activity, effective against bacteria candida and viruses like HSV-1 and HSV-2 [30,31,32,33].

Natural products have gained growing attention for their potential in preventing and treating neurodegenerative diseases. Olive polyphenols, especially oleuropein, have shown protective effects in in vitro and animal studies by reducing amyloid aggregation and promoting the formation of non-toxic oligomeric intermediates [34,35]. Oleuropein enhances the non-amyloidogenic pathway by facilitating amyloid precursor protein (APP) clearance, inhibiting amyloid-beta (Aβ) aggregation, and disrupting preformed Aβ fibrils, positioning it as a promising therapeutic agent for Alzheimer’s disease [36,37,38]. Moreover, biophenols, like oleuropein, exhibit neuroprotective properties through the inhibition of acetylcholinesterase (AChE), butyrylcholinesterase (BChE), and lipoxygenase (LOX), crucial targets in AD treatment [39,40]. Animal studies further validate oleuropein’s neuroprotective potential, linking it to an increased lifespan and improved cognitive function [41]. In a mouse model, a 3-month OLE-enriched diet slowed Alzheimer’s progression by reducing neuroinflammation, promoting anti-inflammatory pathways, and enhancing amyloid clearance [42]. Another study using an extract of roasted date seeds, nigella, and olive oil showed hippocampal regeneration and cognitive improvement in AD rodents [43].

Most research has focused on extra virgin olive oil (EVOO), rich in biophenols, like oleuropein and hydroxytyrosol, showing its ability to reduce tau pathology, enhance brain plasticity, improve memory, restore mitochondrial function, and protect neural cells from amyloid aggregation [44,45,46]. In stroke models, hydroxytyrosol (HT) improved memory, reduced neuroinflammation, and increased cerebral blood flow [47], while in AD mice, HT improved neuronal viability and activity [48]. Olive oil has demonstrated neuroprotective properties also in Parkinson’s disease (PD). In a worm model of PD, olive oil enhanced locomotion, reduced α-synuclein accumulation, and protected dopaminergic neurons from neurodegeneration [49]. Oleuropein also shows promise for multiple sclerosis (MS) by mitigating oxidative stress, upregulating antioxidant enzymes, and preserving myelin integrity [50]. Furthermore, in an epilepsy rat model, OLE significantly reduced the seizure score and oxidative stress index while increasing the level of glutathione [51].

Numerous human trials involving the Mediterranean diet (MeDi) and extra-virgin olive oil (EVOO) support the beneficial effects of biophenols on brain function. The MeDi’s high antioxidant content decreases the subjects’ likelihood of developing AD [52]. Additionally, adherence to the MeDi is linked not only to a lower risk of developing AD and mild cognitive impairment (MCI) but also to a reduced likelihood of MCI progressing to AD. Clinical trials have demonstrated that greater adherence to the MeDi is associated with improved cognitive performance, as measured by the Mini-Mental State Examination (MMSE) and Brief Cognitive Screening Battery (BCSB) [53,54,55]. The MICOIL study in Greece showed that long-term EVOO consumption significantly improved cognitive function, even in individuals with the APOE4 gene variant, which is associated with increased AD risk [56]. Similarly, the MedLey study found that a MeDi enriched with EVOO enhanced cognitive performance, particularly in processing speed, memory, attention, and executive function, as well as psychological well-being in adults over 65 [57]. Moreover, EVOO has shown promising results in treating severe depression, with daily consumption leading to a significant improvement in depression scores, measured based on the Hamilton Depression Rating Scale [58].

In conclusion, olive leaves exhibit beneficial effects on various age-related diseases, largely due to their anti-inflammatory properties. For chronic conditions, like AD, where current treatments are insufficient, discovering complementary therapies is essential. Olive leaf extract (OLE), when used alongside standard AD treatments, may offer additional protection. While laboratory and animal studies support OLE’s potential, human trials assessing its impact on cognitive impairment are still lacking.

### Aim of the Study

This is the first reported randomized clinical trial that administered Greek olive leaf extract to people with mild AD. Our experimental study’s objective was to compare the effects of Greek OLE in combination with the Mediterranean diet (Group 1) versus only the Mediterranean diet (Group 2), on the cognition of patients with mild AD. The duration of the research was six months. The trial involved elderly Greek-speaking participants between the ages of 55 and 85, who lived in the community and were members of the Greek Association of Alzheimer’s Disease and Related Disorders (GAADRD). Olive leaves were selected for their potential health benefits, such as their antioxidant, anti-inflammatory, and antimicrobial properties, as well as their high concentration of polyphenols in comparison to virgin olive oil. Furthermore, numerous in vitro studies have demonstrated OLE’s ability to decrease the aggregation of Aβ42 and tau proteins. The olive leaves utilized in the trial were sourced from award-winning olive trees in the Mediterranean area (Halkidiki of Macedonia, Greece) in 2020. The Golden Tree Company (Halkidiki, Greece) provided the leaves for free without burden. The hypothesis was that participants in Group 1 would demonstrate an improvement in cognitive function, as determined by a neuropsychological assessment after six months, in comparison to participants in Group 2.

## 2. Materials and Methods

### 2.1. Beverage of Greek Olive Leaves

The olive leaves used in this study were sourced from olive trees in Halkidiki, supplied by the Golden Tree Company, and were certified organic by the Hellenic Ministry of Rural Development and Food, with a code number GR-BIO-12. Prior to participant recruitment, the leaves were analyzed for pesticide and phthalate ester concentrations and were found to contain less than 0.01 mg/kg and 0.10 mg/kg, respectively, making them acceptable for use in a clinical trial. These leaves are known to contain high levels of oleuropein, the most important bio-phenolic compound, which has various health advantages in the treatment of numerous chronic diseases [59]. The oleuropein concentration in the leaves was measured using the High-performance Liquid Chromatography with Photodiode-array Detection technique (HPLC-DAD) at 280 nm and was found to be between 1.98 (±0.09) and 3.96 (±0.09) g/100 g dry olive leaves, according to our most recent chemical analysis conducted in 2020 and 2021. Having been dried and chopped, olive leaves were extracted with methanol for 30 min under irrigation and a 60 °C temperature. After the filtration procedure, the methanolic extracts were diluted with water and analyzed for their phenolic compounds’ quantity. Including estimations of pH, CaCO_2_, NO_3_, P, Na, K, Ca, Mg, B, Zn, Fe, Mn, and Cu concentrations, all were tested and found to be within acceptable ranges. Furthermore, the pesticides were examined in depth, and certificates were issued attesting to the high quality of the soil’s components, (extra virgin) olive oil and olive leaves. Participants in Group 1 were asked to prepare a daily beverage using 21 g of chopped, dry olive leaves steeped in 450 mL of room temperature water for 20 min. This allowed consumers to access the healthful components of olive oil without consuming excessive quantities and thereby limiting their caloric intake [12].

### 2.2. Mediterranean Diet (MeDi)

Numerous studies have demonstrated that the health benefits of the Mediterranean Diet (MeDi) are well-known. People with higher rates of MeDi adherence have lower rates of coronary heart disease and cancer-related mortality, as well as lower risks of dyslipidemia, hypertension, abnormal glucose metabolism, obesity, cerebrovascular disease, stroke, depression, and cognitive impairment [60,61,62]. Therefore, an increasing number of individuals attempt to incorporate MeDi into their way of life. It consists of a nutritionally balanced diet. A food pyramid is frequently used to illustrate the proportions of individual food categories’ consumption. Fruits, vegetables, whole grains, and olive oil for cooking and salads are recommended for daily consumption at the base of this pyramid. In the middle, which should be consumed at least twice per week, are legumes, fish, nuts, and poultry. Moreover, a moderate consumption of dairy products such as cheese and yogurt is recommended. At the top of the pyramid are saturated fats and meat (especially red meat), both of which should be consumed sparingly (two to four times per month). Wine (mostly red) is also ingested in moderation with meals, a fact that is currently disputed by some scientists.

### 2.3. Participant Recruitment

The study recruited participants from two Day Centers of the Greek Association of Alzheimer’s Disease and Related Disorders (GAADRD) between September 2020 and December 2021. Participants in the study were diagnosed with mild AD by an expert neuropsychiatrist. A thorough laboratory examination was conducted, which included general blood tests, hepatic and renal biochemical tests, tests for thyroid-stimulating hormone (TSH), free triiodothyronine (FT3), free thyroxine (FT4), vitamin B12, vitamin D, folic acid, homocysteine, and a syphilis screening (RPR). Brain imaging, primarily through magnetic resonance imaging (MRI), or, in cases of patient objections, computed tomography (CT), was conducted to exclude secondary or reversible causes of dementia. After completing the medical examinations, patients underwent detailed neuropsychological testing. Based on the neuropsychological results, medical history, and clinical examinations, the neuropsychiatrist made the final diagnosis. Participation in the study was completely voluntary. All participants signed a consent form containing all trial-related information, which was explained to them orally and written. They were assured that they could withdraw from the research project without repercussions at any time. The Ethical Committee of GAADRD approved the study, known as the GOLDEN study, in September 2020 (approval code 63/12 December 2020), in accordance with the Declaration of Helsinki. The study was registered as a clinical trial under the registration number NCT04440020.

### 2.4. Inclusion Criteria

Participants in the study were between 55 and 85 years old, with a confirmed diagnosis of mild Alzheimer’s disease (AD) and a minimum educational level of five years. All subjects were receiving Donepezil, a cholinesterase inhibitor, with a stable dosage for at least four months prior to enrollment. Additionally, participants were on stable treatments for other chronic conditions, except those outlined in the exclusion criteria. This ensured consistency in their medical management and minimized potential confounding factors that could affect the study outcomes. The score of the Geriatric Depression Scale (GDS) was under ten and the score of the Hachinski Modified Ischemic Scale was equal or under four.

### 2.5. Exclusion Criteria

Subjects were excluded due to the following:

1. Inadequate auditory and visual acuity for neuropsychological testing;
2. Participation in other trials or studies;
3. History of other neurological disorders, such as epilepsy, multiple sclerosis, stroke, or severe psychiatric disorders like schizophrenia, major depression, or severe anxiety disorder.
4. Use of forbidden medications:
  (i) Antidepressants with anti-cholinergic properties, such as tricyclic antidepressants, paroxetine.
  (ii) Frequent use of opioid analgesics (greater than two doses per week) within four weeks preceding screening.
  (iii) Utilization of neuroleptics with anticholinergic actions within four weeks of screening (e.g., clozapine, risperidone, olanzapine, chlorpromazine).
  (iv) Chronic prescription of other medicines with significant anti-cholinergic effects on the central nervous system within four weeks of screening (e.g., H1 receptor antagonists, first-generation orphenadrine, or tizanidine)
  (v) Use of antiparkinsonian treatment within 4 weeks of screening

### 2.6. Neuropsychological Assessment

The neuropsychological evaluation focused on assessing key cognitive domains, including attention, working memory, episodic memory, visuospatial skills, executive functions, and language abilities, providing a broad overview of the participants’ cognitive status. The neuropsychological tests included the following:

1. The Mini-Mental State Examination (MMSE) to evaluate the general cognitive function or Hindi Mental State Examination test in cases of participants with a low level of education. An unauthorized version of the Greek MMSE was used by the study team without permission. The MMSE is a copyrighted instrument and may not be used or reproduced in whole or in part, in any form or language, or by any means without written permission of PAR.
2. Global Clinical Dementia Rating score (CDR) for general cognitive and functional conditions.
3. Alzheimer Disease Assessment Scale-Cognitive (ADAS-Cog) to assess the severity of cognitive dysfunction.
4. Functional Cognitive Assessment Scale (FUCAS), for daily functioning assessment.
5. Functional Rating Scale for Dementia (FRSSD), to evaluate daily functioning.
6. Geriatric Depression Scale for depressive symptoms (GDS).
7. Neuropsychiatric Inventory for the evaluation of other psychiatric symptoms (NPI).

### 2.7. Study Design and Procedure

**Figure 1:**
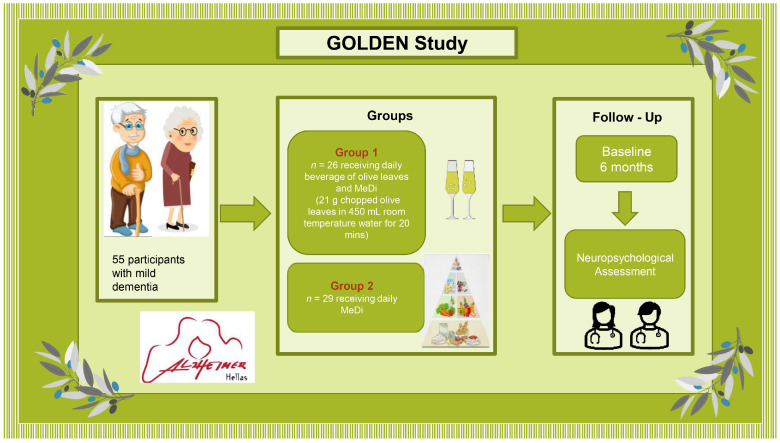
Initial plan for the Golden Study after recruitment and randomization of participants, allocation to Groups 1 and 2, and neuropsychological evaluation six months later.

Each patient at the Day Center was evaluated by a psychologist employing specialized neuropsychological tests. The patient was then examined by a neuropsychiatrist who makes a diagnosis and determines if the patient met the inclusion criteria based on demographic information, medical history, neuropsychological assessment, neurological and laboratory examinations, and MRI or CT brain scans. To be eligible for the study, all patient examinations had to b be current, with laboratory tests and neuropsychological evaluations performed within the past six months and MRI or CT scans performed within the last twelve months. Once a patient met all eligibility requirements, a health professional informed them of the study, explained the procedures, and obtained their written consent. Figure 1 illustrates the study design, including the randomization of participants with mild AD into two groups. Each participant was assigned to one of two groups at random: Group 1 received olive leaves and a recipe to make a daily olive leaf extract (OLE) beverage as well as Mediterranean Diet (MeDi) instructions, while Group 2 received only the MeDi instructions. The minimum duration of the study was six months.

Participants in Group 1 were required to visit the Day Center monthly, either alone or with a caregiver, to receive a 30-day supply of olive leaves. During these visits, a healthcare professional conducted brief interviews to assess adherence to the prescribed diet and monitor any adverse events or trial-related complications. Each participant in Group 1 consumed the same daily dose of olive leaf extract (OLE). They were instructed to prepare a daily beverage by steeping 21 g of chopped, dried olive leaves in 450 mL of room-temperature water for 20 min. After removing the leaves, participants could consume the OLE beverage either all at once or divided into two to three portions throughout the day. The total daily intake of OLE remained consistent for all participants in Group 1.

In contrast, Group 2 participants were not required to attend regular visits but participated in monthly semistructured phone interviews with a health professional to monitor their adherence to the Mediterranean Diet (MeDi). The MeDi Scale we used was developed by Panagiotakos et al. and has been widely used in research to investigate the health benefits and associations of MeDi with various outcomes, such as cardiovascular diseases, diabetes, and overall mortality [61]. The MeDi Scale assigns points to various components of the MeDi based on an individual’s consumption. The components typically considered in the score include the following: (1) fruits, a higher consumption of fruits is associated with a higher score; (2) vegetables, similarly, a higher consumption of vegetables contributes to a higher score; (3) legumes, the consumption of legumes, such as beans, lentils, and chickpeas, is positively scored; (4) cereals, cereals and whole grains are included in this category and are recommended for daily consumption; (5) fish, the regular consumption of fish, which is a good source of omega-3 fatty acids, is associated with a higher score; (6) meat and meat products, lower consumption of meat and meat products is favored in the Mediterranean diet, so higher intake leads to a lower score; (7) dairy products, the MeDi score considers the moderate consumption of dairy products, such as milk, cheese, and yogurt; (8) alcohol, moderate alcohol consumption, typically a glass of red wine during meals, is positively scored; (9) ratio of monounsaturated to saturated fatty acids, a higher ratio of monounsaturated to saturated fatty acids corresponds to a higher score. By assigning points to each component, the MeDi Score provides a quantitative measure of adherence to the MeDi. Higher scores indicate greater adherence, while lower scores suggest poorer adherence to the diet. The scoring methodology devised by Panagiotakos et al. for each category of the MeDi Score ranges from 0 to 5 based on the frequency of consumption. The final score ranges from 0 to 55, with calculated tertiles representing low (0–20), moderate (21–35), and high (36–55) adherence to the MeDi.

#### 2.7.1. Randomization

After the baseline assessment, patients with mild AD who met all the inclusion criteria and gave consent to participate in the clinical trial, were randomized to one of the two groups. For the randomization, we used the RAND function in Microsoft Excel 2010. This function is used to generate a random number between 0 and 1. In human studies, it can be used to randomly assign participants to different groups or conditions or to randomize the order in which stimuli are presented. The result will be a random number between 0 and 1. It is important to note that Excel’s RAND function is a pseudo-random number generator, meaning that the generated numbers are not truly random but are instead generated using a mathematical algorithm, which is called the Mersenne Twister algorithm, and it is designed to generate a long sequence of high-quality pseudo-random numbers that are statistically indistinguishable from true random numbers. It is one of the best and most efficient algorithms for generating pseudo-random numbers and is widely used in various programming languages and applications. However, for most applications, Excel’s RAND function is adequate for generating random numbers.

#### 2.7.2. Participants’ Withdrawals and Abandonment

**Figure 2:**
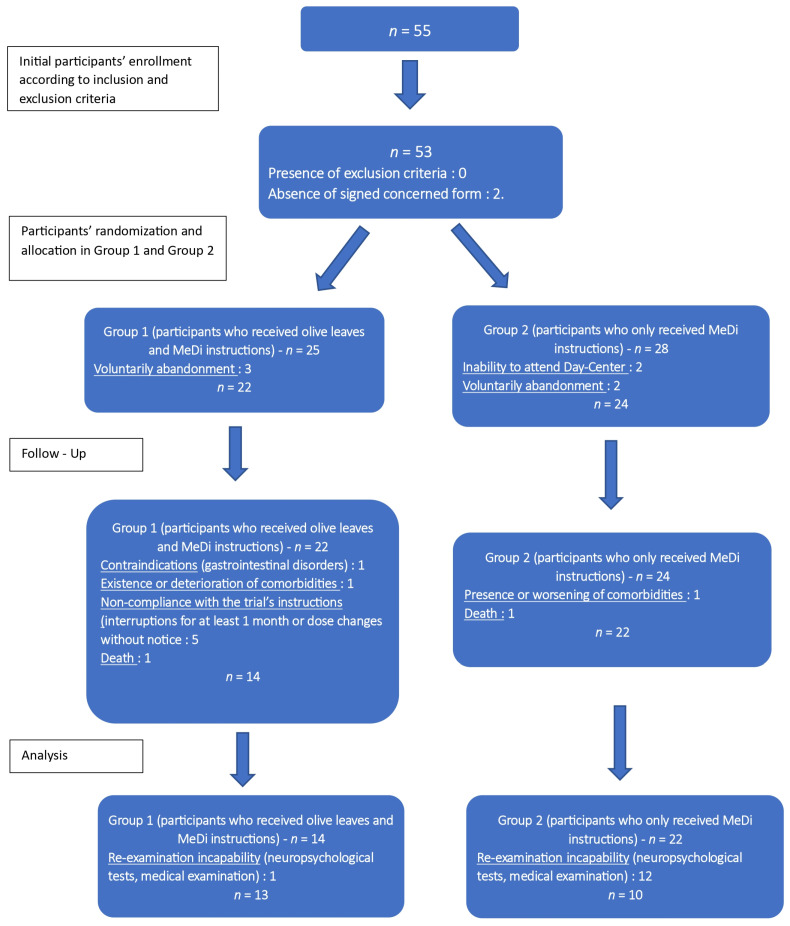
Flow chart presenting participants’ enrollment, randomization, allocation in Groups 1 and 2, withdrawals during the procedure, and the final sample included in the statistical analysis.

The only person who knew who was in each group until the conclusion of the six-month follow-up was the project manager, who was also in charge of distributing the olive leaves and providing MeDi instructions monthly. Neither the health professionals nor the patients themselves could choose which group they would be divided into, as this was determined by the randomization system. Therefore, all independent evaluators were blind to the group assignment. The interviews and neuropsychological evaluation were conducted in a manner that concealed the participants’ allocation. N = 55 patients were initially enrolled in the study and randomly assigned to one of the two groups; however, only 23 patients completed the study (Figure 2).

Situations responsible for participants’ withdrawal were as follows: (1) death or severe medical issues, such as heart or kidney failure, that manifested during the procedure; (2) a participant’s refusal to sign a consent form; (3) presence of adverse side effects after consuming OLE, mainly gastrointestinal disorders; (4) one participant withdrew voluntarily from the study for personal reasons; (5) failure to adhere to the trial’s instructions; (6) intervention with OLE or MeDi guidelines lost for more than a month; (7) some denied assessment after the six-month follow-up.

There were 32 withdrawals from the study: 12 in Group 1 and 18 in Group 2, with reasons outlined (Figure 2). The majority of participants who did not complete the study cited difficulties in attending the monthly visits to the GAADRD Day Centers and completing the comprehensive neuropsychological assessments, laboratory tests, and the final medical evaluation by the expert neuropsychiatrist. This discontinuation was primarily due to the COVID-19 pandemic, which heightened fear, particularly among the elderly, and discouraged them from leaving their homes to attend the Day Centers.

#### 2.7.3. Statistical Analysis

All statistical analysis was performed with R studio, a programming language for statistical computing and graphics. The aim of our study was to evaluate the neuroprotective effects of olive leaves by comparing changes in neuropsychological assessment scores between the two groups. We measured the percentage change in scores before and after the intervention and contrasted these results with those of Group 2, which did not consume olive leaves. To achieve this, we created a new variable for each neuropsychological test, representing the difference between the initial and final scores. For example, “MMSE_dif” calculated the difference in MMSE scores at the beginning and after six months of intervention. We then compared this variable between the two groups to determine if there were statistically significant differences. This procedure was repeated for all neuropsychological tests, generating a difference variable for each test and conducting statistical analyses to compare outcomes between Group 1 and Group 2.

To proceed with the statistical analysis of categorical variables (gender, cases, or controls) and express their results as percentages, we used the chi-square test or Fisher’s test, the latter being an alternative test for situations where the chi-square test conditions were not met or because of our small sample size Fisher’s test led to more accurate conclusions. For continuous variables (age, years of education, neuropsychological assessment), we utilized the student *t*-test when variables were normally distributed, and their results were expressed as the mean and standard deviation, or the Mann–Whitney–Wilcoxon U-test when variables were not normally distributed, and their results were expressed as the median and interquartile range. For contractions between case and control groups, we utilized the independent *t*-test, which compares the mean of the differences between the initial and final value for every neuropsychological test in the two groups, or the Wilcoxon Signed Rank test for non-parametric variables, with the Bonferroni correction. Normality was examined graphically with boxplots or histograms and arithmetically with the Shapiro test and the Levene’s test, when necessary, for investigating the variances’ equality. Lastly, it is generally accepted that *p*-values less than 0.05 are statistically significant.

## 3. Results

In terms of demographic characteristics, including age (years), education (years), and gender distribution (F/M), no statistically significant differences were found between the two groups.

Regarding baseline neuropsychological assessments, conducted prior to study enrollment, most tests revealed no significant differences between the groups. The only exception was the Functional Cognitive Assessment Scale (FUCAS), where scores were significantly higher in the OLE group, suggesting reduced daily functioning compared to controls. Other assessments, including the Mini-Mental State Examination (MMSE), Alzheimer’s Disease Assessment Scale-Cognitive Subscale (ADAS-Cog), Clinical Dementia Rating (CDR), and the Functional Rating Scale for Symptoms of Dementia (FRSSD), showed no statistically significant differences between the two groups. In terms of neuropsychiatric symptoms, both the Neuropsychiatric Inventory (NPI) and Geriatric Depression Scale (GDS) indicated no significant differences between the groups.

Table 1 presents the initial values of all neuropsychological tests. Except for the GDS, all other tests followed a normal distribution. Similarly, Table 2 displays the results of neuropsychological assessments after a 6-month follow-up, where all variables, except for the NPI, were normally distributed, with *p*-values greater than 0.05 in both groups, as determined by the Shapiro–Wilk test. A full breakdown of statistical analyses, including the Shapiro–Wilk test for normality, Levene’s test for equality of variances, and comparative tests (independent *t*-tests and Wilcoxon rank sum tests), is provided in the Supplementary Materials (Tables S1 and S2).

To better capture the longitudinal effects of the intervention on cognitive, functional, and psychiatric outcomes, we created a set of difference variables (_dif) for each primary neuropsychological measure. These difference variables (MMSE_dif, ADAS_dif, CDR_dif, FRSSD_dif, NPI_dif, GDS_dif, and FUCAS_dif) were calculated by subtracting the follow-up scores from the baseline scores. This approach enabled us to focus on individual changes over time, which is crucial when assessing interventions aimed at slowing or reversing disease progression.

The analysis of MMSE_dif, the variable representing the change in MMSE scores from baseline to 6 months, revealed a notable difference between the two groups. Participants in Group 1 (receiving OLE in addition to MeDi) exhibited significantly less cognitive decline compared to those in Group 2 (controls receiving only MeDi instructions). The mean change in MMSE scores for Group 1 was close to zero, indicating stability in cognitive function over the study period. In contrast, Group 2 experienced a marked decline in MMSE scores, with a mean change of approximately −4 points. This significant difference (*p* < 0.05) suggests a potentially protective role of OLE in maintaining cognitive function over time.

For functional measures, the FRSSD_dif variable, representing changes in the Functional Rating Scale for Symptoms of Dementia, showed a modest improvement in Group 1 compared to Group 2. Specifically, participants in Group 1 experienced a slight reduction in FRSSD scores, suggesting a potential improvement in daily functioning, whereas Group 2 saw an increase in FRSSD scores, indicating a decline in their ability to perform daily tasks. Although these changes favored the OLE group, the differences were not statistically significant. Similarly, the FUCAS_dif variable, capturing changes in the Functional Cognitive Assessment Scale, showed no significant difference between groups. However, Group 1 demonstrated a slight improvement in daily cognitive functioning, while Group 2 experienced a minimal change. The Clinical Dementia Rating (CDR) assessments showed better results in the OLE group, although these differences were not statistically significant. Additionally, while the Alzheimer’s Disease Assessment Scale-Cognitive Subscale (ADAS-Cog) results indicated a worse decline in the control group, the findings were not taken into account due to a significant amount of missing data, limiting the ability to draw definitive conclusions from this measure.

In terms of psychiatric symptoms, the NPI_dif variable, representing changes in the Neuropsychiatric Inventory, showed a trend toward improvement in Group 1 compared to Group 2, with the OLE group showing lower NPI scores at follow-up. This indicates a possible beneficial effect of OLE on neuropsychiatric symptoms, although the results did not reach statistical significance. Regarding mood, the GDS_dif variable, which tracked changes in depressive symptoms, showed a minor improvement in the control group compared to the OLE group, but no significant differences were observed between the two groups. It is important to note that depressive symptoms were mild at baseline in both groups.

**Figure 3:**
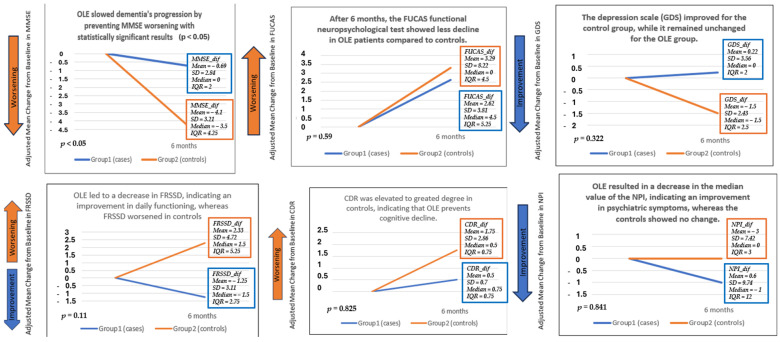
This Figure illustrates the mean difference between the initial and final values of each neuropsychological test for Group 1 (OLE) and Group 2 (controls). For NPI, the difference is represented by the median due to the absence of a normal distribution and the significant number of incomplete data. The ADAS-Cog test had >70% of its data absent, so it could not be evaluated safely. Each group’s mean, standard deviation (SD), median, and interquartile range (IQR) are presented for each variable. OLE led to improvements in memory, cognitive, and functioning neuropsychological tests: MMSE, FUCAS, FRSSD, and CDR, with MMSE results being statistically significant (*p* < 0.05 and Confidence Interval: from 0.779 to 6.04, without including 0). OLE did not appear to help the depression scale (GDS), but it did improve psychiatric symptoms (NPI).

Table 3 presents the mean and standard deviation for variables that followed a normal distribution, along with the median and interquartile range for those that did not, all accompanied by their respective *p*-values. Among these variables, only the MMSE_dif demonstrated a *p*-value of less than 0.05, indicating a statistically significant result. Figure 3 provides a graphical representation of these findings, facilitating a visual comparison of the outcomes between the two groups.

In summary, the most notable outcome was the significant improvement in cognitive function, as measured based on the MMSE, in the OLE group compared to controls. Functional and psychiatric outcomes also showed a trend toward improvement in the OLE group, but these changes were not statistically significant. Detailed statistical analyses for all variables, including normality checks, variance equality tests, and specific statistical comparisons, are provided in the Supplementary Materials.

Table 3 provides a summary of all variables, including demographic information and differences in neuropsychological test scores between the case and control groups following the 6-month clinical trial. For normally distributed variables (MMSE, FRSSD, GDS), the statistical analysis included the mean and standard deviation (SD), whereas it included the median and interquartile range (IQR) for non-normally distributed variables (age, education, FUCAS, CDR, ADAS-Cog, NPI). Statistically significant differences between the two groups were estimated using the independent *t*-test and Wilcoxon rank sum test, with the *p*-values depicted in the final column. The MMSE scores of the group receiving OLE were significantly improved compared to those of the control group (*p* < 0.05), indicating a possible neuroprotective effect of olive leaves on cognitive performance.

Figure 3 illustrates the mean difference between the initial and final values of each neuropsychological test for Group 1 (OLE) and Group 2 (controls). For NPI, the difference is represented by the median due to the absence of a normal distribution and the significant amount of incomplete data. The ADAS-Cog test had >70% of its data absent, so it could not be evaluated safely. Each group’s mean, standard deviation (SD), median, and interquartile range (IQR) are presented for each variable. OLE led to improvements in memory, cognitive, and functioning neuropsychological tests: MMSE, FUCAS, FRSSD, and CDR, with MMSE results being statistically significant (*p* < 0.05 and confidence interval: from 0.779 to 6.04, without including 0). OLE did not appear to help the depression scale (GDS), but it did improve psychiatric symptoms (NPI).

## 4. Discussion

Dietary supplements derived from natural substances have been intensively investigated for their cognitive-enhancing properties. Extra virgin olive oil is integral to the Mediterranean diet, especially in Greece. Olive oil ingredients may improve memory, attention, and general function in patients with mild cognitive impairment or even Alzheimer’s disease, according to studies [17,56,63,64,65]. In addition, many scientists support the theory that mitochondrial dysfunction at the cellular level is responsible for cognitive impairment. Neuronal mitochondrial damage has been linked to oxidative stress. Consequently, the antioxidant properties of olive leaves may aid in maintaining mitochondrial stability and delaying or halting cognitive decline [66].

Olive leaves have never been examined in a human trial so far. The Golden Study is the first randomized clinical trial in humans to test the potential therapeutic properties of olive leaf extract, which may act as a complementary treatment to cholinesterase inhibitors. In our study, the consumption of OLE was associated with improved general cognitive performance after six months of testing, compared to the control group that received only MeDi instructions. The olive leaves provided to the participants for making the daily beverage originated from a single location in Halkidiki. Scientists in the Laboratory of Pharmacy at the National and Kapodistrian University of Athens determined that they are a rich source of oleuropein. Specifically, according to their results, 100 g of dried olive leaves contain 2.96 ± 0.09 g of oleuropein.

Our findings indicate that supplementation with an olive leaf beverage resulted in a statistically significant improvement in the Mini-Mental State Examination (MMSE), the most widely used neuropsychological test for assessing general cognitive function. Participants who consumed olive leaf extract (OLE) for at least six months exhibited stable cognitive performance on average, with a mean difference in MMSE scores before and after the trial approaching zero. In contrast, the control group, which received only Mediterranean diet (MeDi) instructions, showed a statistically significant decline in MMSE scores, with a mean decrease of four units after six months. Furthermore, while the difference in Functional Rating Scale for Symptoms of Dementia (FRSSD) scores was not statistically significant, there was a discernible trend. The OLE group exhibited a mean decrease of −1.3 in FRSSD scores, indicating functional improvement following the six-month intervention, while the control group experienced a mean increase of 2.3, suggesting a decline in functional abilities. This outcome implies that OLE may contribute to the protection of memory, cognition, and daily functioning. Additionally, other assessments, including the Functional Activities Questionnaire (FUCAS) and the Clinical Dementia Rating (CDR), showed better outcomes in the OLE group compared to controls, although these differences were also not statistically significant. Although the Alzheimer’s Disease Assessment Scale-Cognitive Subscale (ADAS-Cog) indicated better outcomes for the OLE group regarding the median difference between initial and final values, a substantial amount of missing data prevented its inclusion in our conclusions. Beyond cognitive assessments, OLE also improved neuropsychiatric symptoms, as demonstrated based on the Neuropsychiatric Inventory (NPI) test. The only unchanged variable was the Geriatric Depression Scale (GDS) for depression, which remained stagnant throughout the study. It is possible that extending the study duration beyond six months, perhaps to one or two years, or increasing the sample size from 55 participants to 100 could yield statistically significant improvements across all variables.

We recommend future longitudinal studies incorporating biomarkers like amyloid, total tau, and phosphorylated tau (P-tau) to determine if olive leaf extract (OLE) can alter the progression of Alzheimer’s disease (AD) pathology. Recent studies on extra virgin olive oil (EVOO) in patients with mild cognitive impairment (MCI) demonstrated that one year of consistent EVOO consumption significantly reduced oxidative and nitrative stress, decreased DNA damage, and restored Alzheimer’s biomarkers (Aβ40, Aβ42) to levels seen in healthy individuals [67,68]. These findings suggest that EVOO may help prevent the progression from MCI to AD. Additionally, EVOO’s antioxidant properties have been shown to restore the AD-associated proteins p-tau and amyloid Aβ, supporting its role in preventing disease progression [69]. High-phenolic EVOO also improved neurophysiological functions in MCI patients by reducing the theta/beta ratio in EEG readings and enhancing functional connectivity and blood–brain barrier (BBB) integrity as demonstrated by MRI studies [70,71]. Considering that olive leaves contain a significantly higher concentration of oleuropein and hydroxytyrosol compared to olive oil—oleuropein concentrations in olive leaves range from 1% to 14%, compared to just 0.005% to 0.12% in olive oil—OLE may potentially have even greater neuroprotective effects [72]. Therefore, OLE could surpass olive oil in preventing AD progression due to its superior biophenol content.

Future randomized studies incorporating olive leaf extract (OLE) with larger sample sizes, additional biomarkers, and longitudinal cognitive assessments are necessary to establish stronger evidence of its neuroprotective role when consumed as a daily beverage. Given the low manufacturing costs and abundance of olive leaves in the Mediterranean region, OLE presents a practical and affordable supplement for enhancing cognitive function in patients. The process of producing OLE is relatively inexpensive, especially when considering the simplicity of providing harvested olive leaves for daily consumption. This is particularly relevant for elderly populations, who often face economic challenges and are already burdened with multiple medications. Considering the growing economic and social burden posed by dementia and cognitive impairment, it is crucial to explore natural products, especially those that are abundant and currently underutilized. Sharing these research findings can help make affordable and effective treatments accessible to a broader population.

For our next steps, we plan to expand the study by increasing the number of participants and extending the duration to evaluate whether the observed results hold or improve over time. Additionally, we aim to introduce a third group consuming a higher dose of OLE to explore any potential dose-dependent effects on brain function through neuropsychological evaluations. Investigating the impact of olive leaf extract on patients’ blood profiles using proteomics, lipidomics, urine, and CSF biomarkers, or even tracking changes in the gut microbiome, would be a valuable yet challenging direction for our future research.

## 5. Conclusions

The GOLDEN study demonstrated that the daily consumption of an olive leaf extract for six months led to a significant improvement in all neuropsychological tests evaluating memory, cognitive performance, and daily functioning, with the MMSE results being statistically significant (*p* < 0.05). Given a larger sample size or an extended trial duration, the neuropsychological evaluations may yield even more pronounced improvements. Introducing OLE as an accessible and cost-effective dietary supplement for individuals with memory impairment can synergistically enhance existing therapies, ultimately improving cognitive and functional outcomes. This research highlights the potential of natural products in advancing treatment strategies for neurodegenerative conditions and represents a significant opportunity for both the scientific community and patients alike.

### Limitations

The major problem of our study was the small sample size, primarily due to patients’ isolation and discouragement stemming from the COVID-19 pandemic. Despite this limitation, similar human studies investigating the beneficial properties of olive leaf extract (OLE) in other areas, such as immune system health, have successfully utilized comparable sample sizes. Because of our study design, only individuals with mild AD were included. It would be a very interesting approach to test olive leaf extract in people with MCI and to investigate the possible decrease in the percentage of patients who develop AD from MCI compared to the general population with MCI. Of course, other factors, such as the presence of APOE ε4 should also be considered. Another limitation of the study is the short duration of the experiment. We already have some participants who have taken the olive leaf extract for one or two years. A larger study with more researchers and more centers assisting in the study process will follow, which will include more participants taking OLE for at least twelve months and will compare the results with those of the six-month follow-up. Finally, our study unfortunately lacked much data on the outcomes of participants’ neuropsychological assessments. Apart from MMSE results, there are missing data in all the other variables. Each patient had a digital medical envelope that contained all neuropsychological tests, as well as the results of the blood test and radiological examinations. This envelope was uploaded to a large database where anonymity prevailed because each patient’s name was replaced by a unique code and only physicians had access to it. Due to an electronic problem in this web database, some valuable data on the participants’ neuropsychological examination were lost.

## Figures and Tables

**Table 1:** Initial values of the Neuropsychological evaluation in both the case and control groups, as well as statistical analysis with independent *t*-tests for normally distributed variables (Mean and Standard Deviation (SD)) and Wilcoxon rank sum tests for non-normally distributed variables (Median and Interquartile Range (IQR)).

Characteristic	Case, *n* = 13	Control, *n* = 10	*p*-Value
Age, Median (IQR)	77.0 (3.0)	78.0 (7.5)	0.4
Education, Median (IQR)	9.0 (9.0)	7.0 (5.3)	0.5
MMSE_A, Mean (SD)	21.1 (3.9)	22.9 (3.1)	0.3
FRSSD_A, Mean (SD)	7.60 (2.37)	6.17 (2.48)	0.3
Unknown	3	4	
GDS_A, Median (IQR)	2.00 (1.50)	4.00 (4.00)	0.4
Unknown	2	2	
FUCAS_A, Mean (SD)	56.0 (5.9)	48.3 (2.9)	0.007
Unknown	3	3	
CDR_A, Mean (SD)	3.64 (1.91)	3.07 (1.62)	0.7
Unknown	6	3	
ADAS_A, Mean (SD)	32 (11)	26 (7)	0.3
Unknown	3	3	
NPI_A, Mean (SD)	8 (7)	17 (15)	0.5
Unknown	5	4	

Wilcoxon rank sum test or Wilcoxon rank sum exact test was used for statistical analysis.

**Table 2:** Final values of the Neuropsychological evaluation in both the case and control groups, as well as statistical analysis with independent *t*-tests for normally distributed variables (Mean and Standard Deviation (SD)) and Wilcoxon rank sum tests for non-normally distributed variables (Median and Interquartile Range (IQR)).

Characteristic	Case, *n* = 13	Control, *n* = 10	*p*-Value
MMSE_B, Mean (SD)	20.4 (3.7)	18.8 (4.5)	0.4
FRSSD_B, Median (IQR)	7.0 (2.0)	9.0 (8.0)	0.2
Unknown	4	1	
GDS_B, Mean (SD)	3.22 (2.64)	3.25 (2.71)	>0.9
Unknown	4	2	
FUCAS_B, Mean (SD)	57 (7)	56 (10)	>0.9
Unknown	2	0	
CDR_B, Mean (SD)	4.10 (2.61)	4.83 (3.33)	>0.9
Unknown	8	4	
ADAS_B, Mean (SD)	29.8 (9.3)	30.0 (1.4)	>0.9
Unknown	7	6	
NPI_B, Median (IQR)	2 (5)	14 (12)	0.2
Unknown	5	3	

**Table 3:** The table provides a summary of all variables, including demographic information and differences in neuropsychological test scores between the case and control groups following the 6-month clinical trial. For normally distributed variables (MMSE, FRSSD, GDS), statistical analysis includes the Mean and Standard Deviation (SD), whereas it includes the Median and interquartile range (IQR) for non-normally distributed variables (age, education, FUCAS, CDR, ADAS-Cog, NPI). Statistically significant differences between the two groups were estimated using the independent *t*-test and Wilcoxon rank sum test, with the *p*-values depicted in the final column. The MMSE scores of the group receiving OLE were significantly improved than those of the control group (*p* < 0.05), indicating a possible neuroprotective effect of olive leaves on cognitive performance.

Characteristic	Case (*n* = 13)	Control (*n* = 10)	*p*-Value
Age, Median (IQR)	77.0 (3.0)	78.0 (7.5)	0.4
Education, Median (IQR)	9.0 (9.0)	7.0 (5.3)	0.5
MMSE_diff, Mean (SD)	−0.69 (2.84)	−4.10 (3.21)	0.009
FRSSD_diff, Mean (SD)	−1.3 (3.1)	2.3 (4.7)	0.2
Unknown	5	4	
GDS_diff, Mean (SD)	0.22 (3.56)	1.50 (2.43)	0.3
Unknown	4	4	
FUCAS_diff, Median (IQR)	4.5 (5.3)	0.0 (4.5)	0.6
Unknown	5	3	
CDR_diff, Median (IQR)	0.75 (0.75)	0.50 (0.75)	0.8
Unknown	9	8	
ADAS_diff, Median (IQR)	2.5 (6.6)	3.3 (1.2)	0.9
Unknown	10	6	
NPL_diff, Median (IQR)	−1 (12)	0 (3)	0.8
Unknown	8	5	

## Data Availability

Data used in this work are available under request to the corresponding author.

## Supplementary Materials

The following supporting information can be downloaded at: https://www.mdpi.com/article/10.3390/neurolint16060095/s1. The supplementary material section includes a complete statistical analysis for each parameter of the neuropsychological test, along with two additional tables. Table S1 delineates the summary of the statistical analysis concerning the initial values of the neuropsychological tests, encompassing normal distribution, variance equality, and statistical differences, while Table S2 offers a summary of the statistical analysis performed on the final neuropsychological test results.
