# Supplementary material for: The Role of Greek Olive Leaf Extract in Patients with Mild Alzheimer’s Disease (the GOLDEN Study): A Randomized Controlled Clinical Trial
Source: Neurol Int. 2024 Oct 29;16(6):1247–65. doi: 10.3390/neurolint16060095 (PMC11587000; doi:10.3390/neurolint16060095)
Supplement: Supplementary file 1 [file neurolint-16-00095-s001.zip › neurolint-3103529-supplementary.pdf]

neurology international

MDPI

Article

The Role of Greek Olive Leaf extract in patients with mild Alzheimer's disease (the GOLDEN study): a randomized controlled clinical trial

Sofia Loukou [1,2,3]*, Georgia Papantoniou [3,4], Anastasia Pantazaki [3,5] and Magdalini Tsolaki [1,2,3]

1 1st Department of Neurology, Medical School, "AHEPA" General Hospital Medical School, Faculty of Health Sciences, Aristotle University of Thessaloniki, 54124 Thessaloniki, Makedonia, Greece; tsolakim@auth.gr
2 Greek Association of Alzheimer's Disease and Related Disorders—GAADRD, 54124 Thessaloniki, Greece;
3 Laboratory of Neurodegenerative Diseases, Center for Interdisciplinary Research and Innovation (CIRI-AUTH), Balkan Center, Aristotle University, 54124 Thessaloniki, Greece; gpapanto@uoi.gr (G.P.);natasa@chem.auth.gr (A.P.)
4 Laboratory of Psychology, Department of Early Childhood Education, School of Education, University of Ioannina, 45110 Ioannina, Greece;
5 Laboratory of Biochemistry, Department of Chemistry, Aristotle University of Thessaloniki, 54124 Thessaloniki, Greece;
* Correspondence: sofialoukou1996@gmail.com

Supplementary materials

Results

Regarding their demographic data of Age (years), Education (years), and Gender (F/M), no statistically significant differences were observed between the groups.

Finally, 14 (60.87%) of the 23 participants who completed the study were women. Group 1 (participants receiving the OLE and MeDi guidelines) concluded with 13 participants (with women representing 62%), whereas Group 2 (participants receiving only MeDi instructions) concluded with 10 participants (with women representing 60%). The p-value for the gender distribution between the two categories is 0.9, which is greater than 0.05, indicating that no statistically significant difference exists.

Within each group, the Shapiro-Wilk test evaluates the normality of the education variable. The test statistic for the case group is 0.831 with a p-value of 0.0164. The test statistic for the control group is 0.839 with a p-value of 0.0426. Both p-values are less than 0.05, suggesting sufficient evidence to safely reject the normality null hypothesis. Therefore, it is possible that the education variable is not normally distributed across both categories. The Wilcoxon rank sum test contrasts the education variable distributions of the case and control groups. 75.5 is the test statistic, while p-value is 0.522 (> 0.05). So, there is not enough supporting data to draw the conclusion that the education distributions of the two groups differ significantly. The mean education level of the case group was approximately 9.92, with a standard deviation of 4.73. The median education level is 9 and the interquartile range is also 9, whereas the control group's mean education level was 8.3 with a SD of 3.02. The median level of education is 7, and the IQR is 5.25.

Using the Shapiro-Wilk test, we also examined the age distribution between the two groups. The Shapiro test for the case group produced a statistic of 0.805 and a p-value of 0.00788. The p-value is less than 0.05, indicating that the age variable in the case group significantly deviates from a normal distribution. The Shapiro-Wilk test for the control

group produced a statistic of 0.948 and a p-value of 0.641. The p-value is greater than 0.05, indicating that the age variable in the control group follows a normal distribution. Even if one of the two groups has a normal distribution, the independent t-test cannot be used for statistical analysis. The Wilcoxon rank sum test (Mann-Whitney U test) was then utilized to compare the distributions of the age variable between the case and control groups. 51.5 is the test statistic, and the p-value is 0.417. Since the p-value is greater than 0.05, the age distributions of the two groups do not differ significantly. The mean age of the case group is approximately 75 years (mean = 74.8, with SD = 9.74). The median age is 77 and the interquartile range (IQR) is 3, whereas the mean age of the control group is 78 with SD = 7.42. The median age is 78, and the interquartile range is 7.5.

Table 1 displays the initial values of all neuropsychological tests administered to our participants. Except for the GDS (scale for evaluating depression), all other tests are normally distributed; the Shapiro-Wilk test values for all variables in both case and control groups were greater than 0.05. In addition, we utilized Levene's test to examine the variance equality of normally distributed variables. In every instance, the p-value of Levene's test was greater than 0.05, indicating that there is no statistically significant difference between groups 1 and 2 in terms of variance. In addition, we analyzed each neuropsychological test for statistically significant differences between groups using the independent t-test for normally distributed variables and the Wilcoxon rank sum test for GDS. The results of the Shapiro, Levene's, Wilcoxon rank sum, and independent t-test for all neuropsychological tests are presented in Table S1. Except for FUCAS, which was statistically significantly higher in the case group compared to the control group, all P-values were greater than 0.05. This indicates that patients receiving OLE had a lower level of daily functioning than controls. The other functioning neuropsychological test, FRRSD, did not differ significantly between groups, with a mean score of 7.6 in Group 1 and 6.2 in controls. The most prevalent neuropsychological assessment of cognitive status, the MMSE, had mean scores of 21,1 in cases and 22,9 in controls. Another evaluation of memory and cognition, ADAS-Cog, yielded a mean of 31 in cases and 27 in controls, while CDR yielded a mean of 3.5 in cases and 3 in controls. All aforementioned assessments of cognition, memory, and functional ability yielded better scores in the control group compared to the case group, but the differences were not statistically significant (apart from FUCAS).

Table S1. presents the synopsis of the statistical analysis regarding the initial values of the neuropsychological tests. Except for GDS, all variables have normal distributions, with no statistically significant variance differences between cases and controls. For the statistical analysis of normally distributed variables, an independent t-test was utilized, while the Wilcoxon rank sum test (Mann-Whitney U test) was utilized for GDS. FUCAS was the only neuropsychological test with a p-value less than 0.05 that demonstrated statistically significant differences between the two groups.

	**Shapiro-Wilk test (p-value)**	**Levene's test (p-value)**	**Independent t-test with p-value and Confidence Intervals (CI) / Mann-Whitney U test with p-value**
MMSE_A	Group 1: 0.475, Group 2: 0.482	0.722	t-test: −1.2, p:0.242, CI: [−4.97, 1.32]
FRSSD_A	Group 1: 0.230 , Group 2: 0.794	0.783	t-test: −1.15, p:0.269, CI: [−1.23, 4.10]
FUCAS_A	Group 1: 0.378 , Group 2: 0.897	0.161	t-test: 3.2, p<0.05, CI: [2.56, 12.9]
CDR_A	Group 1: 0.696 , Group 2: 0.687	0.708	t-test: 0.604, p:0.557, CI: [−1.49, 2.63]
ADAS_A	Group 1: 0.377, Group 2: 0.973	0.211	t-test: 1.25, p:0.239, CI: [−4.77, 17]

NPI_A	Group 1: 0.162 , Group 2: 0.205	0.053	t-test: −1.49, p:0.161, CI: [−21.5, 4.02]
GDS_A	Group 1 <0.05, Group 2: 0.416	0.427	Wilcoxon test: 34 (non-normally distributed variable)

Checking for normal distribution, variance equality, and statistical differences in initial neuropsychological tests between Group 1 and Group 2 participants.

Similarly, Tables 2 and S2 display the results of neuropsychological examinations after a 6-month follow-up. Except for the NPI test, all variables were normally distributed with a p-value greater than 0.05 in both cases and controls, as determined by the Shapiro-Wilk test for normal distribution. We proceeded with Levene's test to determine whether the case and control groups had comparable variances. ADAS-Cog and FRSSD test variances between the two groups differed significantly on a statistical level. For the statistical analysis of these two variables, as well as the non-normally distributed NPI, we employed the Mann-Whitney U test, whereas for the remaining variables (MMSE, FUCAS, CDR, GDS), we employed the independent t-test. As can be seen in Table S1, there was no distinguishable difference (*p*>0.05) between the two groups across any measured parameter.

Table S2. provides a summary of the statistical analysis conducted on the final neuropsychological test results. Except for NPI, all variables have normal distributions. FRSSD and ADAS-Cog tests suggest statistically significant variance differences between cases and controls (Levene's test had a *p*-value < 0.05). For the statistical analysis of normally distributed variables with no significant variance differences, an independent t-test was used, whereas the Wilcoxon rank sum test (Mann-Whitney U test) was used for NPI, ADAS, and FRSSD. No variables demonstrated statistically significant differences between the two groups; *p*-values were always greater than 0.05.

	**Shapiro-Wilk test (*p*-value)**	**Levene's test (*p*-value)**	**Independent t-test with p-value and Confidence Intervals (CI) / Mann-Whitney U test with p-value**
MMSE_B	Group 1: 0.132, Group 2: 0.416	0.444	t-test: 0.927, *p*: 0.364, CI: [−1.97, 5.14]
FRSSD_B	Group 1: 0.082 , Group 2: 0.321	<0.05	Wilcoxon test: 27, *p*-value: 0.248
FUCAS_B	Group 1: 0.494 , Group 2: 0.07	0.178	t-test: 0.138, *p*: 0.892, CI: [−7.48, 8.53]
CDR_B	Group 1: 0.872 , Group 2: 0.123	0.685	t-test: - 0.401, *p*: 0.699, CI: [−4.88 3.41]
ADAS_B	Group 1: 0.691 , Group 2: 0.125	<0.05	Wilcoxon test: 12, *p* > 0.9
NPI_B	Group 1: *p* < 0.05 , Group 2: 0.368	0.053	Wilcoxon test: 16.5, *p*-value: 0.202
GDS_B	Group 1: 0.276, Group 2: 0.584	0.801	t-test: −0.02, p: 0.983, CI: [−2.79, 2.74]

Checking for normal distribution, variance equality, and statistical differences in the final neuropsychological tests (after the 6-month Follow-Up) between Group 1 and Group 2 participants.

We developed a new variable, MMSE_dif, to quantify the numerical difference between participants' pre- and post-six-month MMSE scores in both groups (group 1: participants given olive leaf extract as a daily beverage in addition to MeDi instructions for their diet; group 2: participants given only MeDi instructions for their diet). We first used the Shapiro test to see whether the two sets of data were regularly distributed. Shapiro test results showed that MMSE_dif followed a normal distribution, thus we checked for variance equality using Levene's test. Levene's test p-value of 0.796 suggests that there is

no statistically significant difference between groups 1 and 2 in terms of their variances. As a result, we find no evidence that the assumed equal variances have been violated. In addition, we compared the two groups' MMSE_dif means using the independent t-test. With a p-value less than 0.05 ($p < 0.05$) and a t-test = 2.7, we found that after the follow-up, the MMSE in the control group were statistically significantly lower than the MMSE in the group of cases. The MMSE_dif mean, standard deviation (SD), median (M), and interquartile range (IQR) in the group of cases were −0.692, 2.84, 0, and 2, whereas in controls were: −4.1, 3.21, −3.5 and 4.25 respectively. The real mean difference is most likely located within the 95% confidence interval (CI: from 0.779 to 6.04) since this interval does not include 0.

Moreover, we constructed the FUCAS_dif variable, which calculates the difference between the Functional cognitive assessment scale (FUCAS) results after the 6-month follow-up and the FUCAS results at baseline. We checked for a normal distribution in both groups using the Shapiro test; the p-value was less than 0.05 in both groups, indicating that there is no normality, so we moved on to the Wilcoxon test. The Wilcoxon rank sum test contrasts the FUCAS_dif variable distributions of the case and control groups. The test statistic is 33, and the p-value is 0.59, which exceeds the significance threshold of 0.05. Consequently, there is not enough convincing proof to conclude that the distributions of FUCAS_dif in the two groups differ significantly. The following are the summary statistics for the FUCAS_dif variable within each group. In the case group, the mean is 2.62, with a SD of 3.81, the median is 4.5, and the interquartile range (IQR) is 5.25. In the control group: The mean is 3.29 with SD 8.22, while the median is 0 with the IQR being 4.5.

FRSSD_dif is a new variable that represents the difference between the Functional Rating Scale of Symptoms of Dementia (FRSSD) results after the follow-up was completed and at baseline. As with all variables, we used the Shapiro test to estimate the normal distribution of this variable in both the case and control groups. The test statistic for the case group is 0.957 (p-value=0.785) while for the control group is 0.954 (p-value=0.775). In both cases, the p-values are greater than 0.05, indicating that the null hypothesis of normality is not rejected. Within each group, the FRSSD_dif variable appears to have a roughly normal distribution. Additionally, we conducted the Levene's Test: Levene's test investigates the homogeneity of variances for the FRSSD_dif variable between the case and control groups. The test statistic has a p-value of 0.272 and is 1.32. So, there is no evidence to support that the two groups have unequal variances (p>0.05). We then conducted the t-test for Independent Samples which contrasts the means of the FRSSD_dif variable for the case and control groups. The difference between the means is estimated to be −3.58, with group 1 (case) having a lower mean than group 2 (control). The test statistic is −1.72, p-value is 0.111 (> 0.05), and Confidence Interval ranges from −8.13 to 0.959. There is no statistically important difference between the two groups' averages, since the p-value is bigger than 0.05. The following are the summary statistics for the FRSSD_dif variable within each group. For the case group, the mean is −1.25 with standard deviation (SD) 3.11, and the median is - 1.5 with interquartile range (IQR) 2.75, while for the control group the mean is 2.33, (SD = 4.72), and the median is 1.5 with IQR being 5.25. FRSSD in the group receiving OLE decreased by 1.25 units, indicating an improvement in executive function, whereas FRSSD in the control group increased by 2.30 units, indicating a decline in executive function. The results were not statistically significant, however.

The variable CDR_dif was established to represent the difference between the final and initial scores on the Clinical Dementia Rating (CDR) test. We assessed the normality of the CDR_dif variable within each group using the Shapiro-Wilk test, which resulted in 0.827 with a p-value of 0.161 for Group 1 and 0.637 with a p-value of 0.00125 for Group 2.

The p-value for the case group is greater than 0.05, indicating that CDR_dif is normally distributed in the group of cases. In contrast, the p-value for the control group is less than 0.05, indicating that the CDR_dif variable in the control group may not follow a normal distribution. Therefore, we used the Wilcoxon rank-sum test, also known as the Mann-Whitney U test, to contrast the distributions of the CDR_dif variable between the case and control groups. The p-value for the test statistic is 0.825, and the value is 10.5. There is insufficient information to state that there is a statistically significant difference between the two groups' distributions (p-value > 0.05). The following are the summary statistics for the CDR_dif variable within each group. The mean, SD, median, and IQR for the case group are 0.5, 0.707, 0.75, and 0.75 retrospectively, whereas for the control group are 1.75, 2.86, 0.5 and, 0.75. In both groups, the mean and median CDR values increased without any statistically significant difference.

Dementia's cognitive symptoms may be quantified in terms of severity using the Alzheimer's Disease Assessment Scale-Cognitive Subscale (ADAS-Cog), a quick neuropsychological test. We created the variable ADAS_dif to estimate the difference between the participants' final scores and their baseline score. We used the Shapiro-Wilk test to determine whether the ADAS_dif variable within each group is normally distributed. The test statistic for the case group is 0.928 (p = 0.483) while for the control group is 0.760 (p=0.0482). The p-values for both categories exceed 0.05, indicating that there is insufficient evidence to reject the null hypothesis of normality. It is important to note, however, that the p-value for the control group is close to 0.05, indicating a possible departure from normality. Therefore, we utilized the Wilcoxon rank-sum test for the statistical analysis of the ADAS_dif variable between the groups due to our limited sample size, the fact that the ADAS-Cog was not completed by all participants, and the presence of some missing data. The test statistic is five, and the associated p-value is 0.857. According to statistical analysis, there is no significant difference between the distributions of the two groups (p-value > 0.05). The following are the summary statistics for the ADAS_dif variable within each group. The case group has a mean of 4.6, SD of 3.56, a median of 2.5, and an IQR of 6.55, whereas the control group has a mean of 2.58, SD of 2.43, a median of 3.3, and an IQR of 1.23. In each group, ADAS-Cog was enhanced. Even though the increase in ADAS_dif was greater in the control group, this difference was not statistically significant.

In addition to neuropsychological tests for cognitive and functional impairment, we examined the potential impact of OLE on the mood of the participants. Geriatric Depression Scale (GDS) is the most used test for estimating depression in GAADRD. Patients with severe depression or other psychiatric disorders were, of course, excluded from our study. Our participants scored less than 10 on the GDS before the start of the investigation. GDS_dif is a new variable we created to estimate the difference between the GDS values at the 6-month follow-up and their initial values. Using the Shapiro-Wilk test, we first evaluated the normality of the GDS_dif variable within every group. The test statistic for the case group is 0.878 with a p-value of 0.149. The test statistic for the control group is 1.00 with a p-value of 1.00. The p-values for both categories exceed 0.05, indicating that the null hypothesis of normality cannot be rejected. In both categories, the GDS_dif variable appears to be approximately normally distributed. Furthermore, we estimated the equality of variance between the case and control groups for the GDS_dif variable using Levene's test, whose result is 0.106 with the corresponding p-value is 0.750. Since the p-value is higher than 0.05, the variances between the two groups do not significantly differ. Therefore, for the statistical analysis, we utilized the Independent Samples t-test, which contrasts the means of the GDS_dif variable for the case and control groups. The estimated mean difference is 1.72, with p-value being 0.322 (> 0.05) and the confidence intervals ranging from −1.89 to 5.34. Since the p-value is over 0.05, we cannot conclude that there is a statistically significant difference between the two

groups' averages. The mean, SD, median, and IQR were all calculated to be 0.222, 3.56, 0, and 2 for the case group, and −1.5, −2.43, −1.50, and −2.5, −1.5, and −2.5, respectively, for the control group. According to our findings, OLE did not appear to enhance the participants' depressive mood; however, this study did not consider a variety of factors that influence people's emotional state.

Finally, we investigated the potential impact of OLE on participants' psychiatric symptoms using the Neuropsychiatric Inventory (NPI). We created the variable NPI_dif to represent the difference between the final and initial NPI values. As with all other variables we've constructed, before proceeding, we checked for normality using the Shapiro-Wilk method. For the case group, the t-test statistic is 0.937 (p-value = 0.643), whereas for the control group, the t-test is 0.732 (p-value = 0.0200). The p-value for the case group is higher than 0.05, suggesting that there is inadequate proof to reject the null hypothesis of normality. However, the p-value for the control group is less than 0.05, indicating that the NPI_dif variable in the control group may not be normally distributed. The Wilcoxon rank sum test (Mann-Whitney U test) contrasts the distributions of the NPI_dif variable between the case and control groups. 14 is the test statistic, and the p-value is 0.841Not enough proof exists to infer that the distributions of the two groups are different, since the p-value is larger than 0.05. The following are the summary statistics for the NPI_dif variable within each group. The case group has a mean of 0.6 with a SD of 9.74 and a median of -1 with an interquartile range (IQR) of 12, whereas the control group has a mean of −3, SD of 7.42, a median of 0 and an IQR of 3. NPI in the group receiving OLE appears to be lower than NPI values in the control group, which indicates a possible beneficial role of OLE in patients' psychiatric symptoms; however, the results are not statistically significant.

Comparing Group 1 (participants receiving OLE as a complementary treatment) to Group 2 (controls receiving only MeDi instructions), the MMSE was statistically significantly improved in Group 1 (participants receiving OLE) than in Group 2 (controls, receiving only MeDi instructions). Specifically, after a 6-month follow-up, the cognitive status of Group 1 participants according to the MMSE appears stable (the mean of MMSE difference tends to be 0), whereas the cognitive status of Group 2 participants decreased considerably, with a mean MMSE difference close to −4 (p < 0,05). As with other neuropsychological tests, FRSSD values improved substantially in OLE receivers compared to controls whose FRSSD test scores increased, signifying a decline in their daily functioning. Statistically, however, the results were not significant. The remaining neuropsychological tests (CDR, FUCAS, and ADAS) that assessed the cognitive and functional status of the participants and NPI, which assessed their psychiatric symptoms, showed better results in OLE group compared to controls but without any statistically significant differences. GDS, which assessed participants' depressive mood, was the only neuropsychological test that did not reveal better results in OLE group; in group 1 the results showed stagnation whereas in group 2 there was an improvement in the depression scale, but without any significant differences between the two groups. Table S2 displays the Mean and Standard Deviation (SD) of normally distributed variables, along with the Median and inter-quartile range (IQR) of non-normally distributed variables.

Using a multivariate linear regression model, it was determined if the variables MMSE_dif and FRSSD_dif, which revealed the most significant speaking differences between the two groups, were significantly associated with their demographic data: gender, years of education, and age.

The intercept (representing the estimated average MMSE_dif when Education, Age, and Gender are all zero) is 18.78817, but without being statistically significant at the 5% level

(p-value = 0.0835). As indicated by the Education coefficient of −0.08446, a one-unit increase in Education is associated with a 0.08446 decrease in MMSE_dif, which does't present any statistically interest (p-value = 0.6670). The coefficient for age is −0.26849, indicating that a one-unit increase in age is associated with a de-crease of 0.26849 in MMSE_dif. This coefficient is marginally significant at the 5% level (p-value = 0.0523), suggesting that Age and MMSE_dif may be related. The coefficient Gender_M = 0.71498 indicates that the male gender is associated with an increase of 0.71498 in MMSE_dif. However, this coefficient lacks statistical significance (p-value = 0.6522). Multiple R-squared = 0.1883, implying that the model explains about 18.83% of the variance in MMSE_dif. Taking into consideration the number of predictors and sample size, the adjusted R-squared value is 0.06014, reducing the percentage from 18.83% to 6.01%. The global F-statistic evaluates the significance of the model's predictors. The p-value of 0.2546 and the F-statistic of 1.46 indicate that the model is not statistically significant at the 5% level.

The intercept (representing the average estimated FRSSD_dif when Education, Age, and Gender are all zero) is −11.7443, but it is not statistically significant at the 5% level (p-value = 0.559). The coefficient for Education is - 0.3466, which indicates that a one-unit increase in Education correlates with a 0.3466 decrease in FRSSD_dif, but without statistical significance (p-value = 0.422). Age has a coefficient of 0.2012, indicating that a one-unit increase in age correlates with a 0.2012 increase in FRSSD_dif. (p-value = 0.426, $p > 0.05$, meaning no statistical significance). The coefficient for Gender_M (representing male gender) is −0.7939, indicating that the male gender is associated with a 0.7939 decrease in FRSSD_dif compared to the female gender. Moreover, this coefficient, however, does not present any statistical interest (p-value = 0.798). Multiple R-squared equals 0.1972, reflecting that the model explains 19.72% of the variance in FRSSD_dif. The inclusion of predictors results in an adjusted R-squared of 0.0437, which does not improve the model's fit and reduces the percentage to 4.37 percent. The global F-statistic evaluates the significance of the model's predictors as an ensemble. The model is not statistically significant at the 5% level in this instance (F = 0.8186, p = 0.5126).

Individual predictors (Education, Age, and Gender) have no significant effect on the outcome variables (MMSE_dif and FRSSD_dif) in both models.
